# Supplementary material for: Predicting nutritional status for women of childbearing age from their economic, health, and demographic features: A supervised machine learning approach
Source: PLoS One. 2023 May 12;18(5):e0277738. doi: 10.1371/journal.pone.0277738 (PMC10180666; doi:10.1371/journal.pone.0277738)
Supplement: S1 File — (DOCX) [file pone.0277738.s001.docx]

# **Supplementary resources**

## **Supplement A. Description of SML algorithms used in the study**

In Supplement A, we provide the descriptions of algorithms and evaluation measures implemented in this study

**Description of SML algorithms**

*Linear Regression*

Linear regression is a basic form of prediction, and some ML techniques are extensions of this traditional approach [1]. This method assumes that the conditional distribution of the outcome given the set of independent variables follows an *n*-dimensional independent linear combination [2]. We set up our base model as

$$BMI = \beta X + \varepsilon$$

where BMI is the body mass index, *X* and$\varepsilon$ indicate the set of regressors and the error term, respectively.

*Logistic Regression*

The logistic regression model is used to model the probabilities for classification problems with two possible outcomes for a given set of predictors. It employs maximum likelihood to estimate parameters and uses the logistic function to compress the output of a linear equation between 0 and 1. The model is presented as follows,

$$\log\left( \frac{p\left( X \right)}{1-p\left( X \right)} \right)= \boldsymbol{X}\beta$$

where *p* is the probability that the individual has certain outcome.

*LASSO Regression*

The least absolute shrinkage and selection operator regression, more widely known by the acronym LASSO regression (LLR), is a special type of linear regression in which additional variables get penalized in the estimation [3]. It has been adopted in recent economics literatures [4–6] as it works as both variable selection and dimensionality reduction. LASSO achieves these two objectives by solving the following Lagrange multiplier problem.

$$argmin_{\beta}\left( y-X^{T}\beta\right)^{2}+\lambda\left| \beta\right|$$

The equation suggests that a penalty is imposed if the absolute coefficients values are far from zero [7]. As $\lambda$, the penalty attached to additional variables, gets bigger, some variables will get $\beta$ values equal to zero [1]. If $\lambda$ goes to infinity, all coefficient values go to zero. This also suggests both variable selection and dimensional reduction as the estimation results would only show the covariates that survived. We apply both linear and logistic regressions with LASSO in our analysis.

*Regression Tree*

Regression tree (RT hereafter) is a popular tree-based algorithm in which the main dependent variable is a continuous variable. The foundation is to divide the data into subsamples and estimate the regression function within the subsamples as the average outcome [2]. The tree is built with recursive binary splitting, which finds a threshold *t* from an independent variable *X_i_* that minimizes the following equation, and $\hat{y}_{R_{1}}$ and $\hat{y}_{R_{2}}$ are the estimated average outcome values from R_1_ and R_2_ respectively [1].

$$\sum_{x_{i}\in R_{1}} (y_{i}-\hat{y}_{R_{1}})^{2}+\sum_{x_{i}\in R_{2}} (y_{i}-\hat{y}_{R_{2}})^{2}$$

$$R_{1}=\{X|X_{i}<t\}, R_{2}=\{X|X_{i}>t\}$$

It can also be regularized to avoid overfitting [8], and it is called pruning. When pruning is applied, a regression tree solves the following problem

$$\sum_{x_{i}\in R_{1}} (y_{i}-\hat{y}_{R_{1}})^{2}+\sum_{x_{i}\in R_{2}} (y_{i}-\hat{y}_{R_{2}})^{2}+|\alpha|T$$

$\alpha$ and $T$ represent the complexity parameter and the size of the tree respectively. If the value of $\alpha$ is bigger, the tree is expected to be smaller and vice versa. The main strengths of this method are the results are easy to interpret, and they provide a graphic visualization of how the covariates interact with each other to predict an outcome. Unlike in linear regression in which interaction between variables must be specified, regression tree establishes the interaction automatically [9].

*Decision Trees*

Decision tree (DT) is also a tree-based algorithm in which the dependent variable is categorical. It can be built from either the information gain based on entropy or the Gini index. We use the latter, which has the following mathematical equation:

$$G\left( p \right)=\sum_{i=1}^{N} p\left( x_{i}\in c \right)\left( 1-p\left( x_{i}\in c \right) \right)$$

where $p\left( x_{i}\in c \right)$ represents the probability that a regressor belongs to a certain class *c*.

A variable with a higher probability value takes the top node of the tree. In other words, a variable with the lowest Gini index has the most observations from a single class [1]. This indicates that a variable with the smallest amount of randomness is the most important in building a tree. Decision trees also share the same strengths as regression trees and can also be regularized to avoid overfitting.

*Random Forest*

Random forest (hereafter RF) is the extension of both regression and decision trees. It is built under a specific algorithm called bootstrap aggregation [10], in which trees are constructed after sampling the dataset with replacement. The sample of observations that are not used during the building process is called the out-of-bag error. These two steps are repeated a finite number of times, and the final model is decided by taking the average of all trees if the outcome is continuous and the majority rule if the dependent variable is categorical. Due to the repetitive nature of the algorithm, the out-of-bag errors are also equivalent to the cross-validation error [8]. Therefore, random forest can be fitted with a single sequence and yield a lower variance [1].

*K-nearest neighbor*

K-nearest neighbor (KNN hereafter) is a memory-based classifier that does not require modeling [8]. The basic structure of this method is that given a data point, it will try to find *k*-training points that are the closest based on Euclidean distance to the specified data point. Then the outcome is classified using the majority vote among the neighbors. This method can also be extended to a regression setting as well [1].

*Adaptive Boosting*

Boosting is an algorithm to improve the performance of simple supervised methods [46], and we adapt adaptive boosting (ADA) developed by Schapire and Freund [11]. The foundation of this method is that a weak classifier is built for each regressor, and the performance is evaluated [12]. Any incorrectly classified data sample will be adjusted sequentially so that the performance can be improved.

*Support Vector Machine*

Support vector machine (SVM hereafter) is an algorithm which can handle both regression and classification. The goal of this algorithm is to find a hyperplane which has the maximum distance between two classes, and units that have the same distance to the hyperplane are the support vectors [1,2]. Given a hyperplane,

$\alpha+\beta^{T}X=0$*,*

the outcome will be classified as 1 if $\alpha+\beta^{T}X\geq0$, and -1 otherwise if the data can be separated linearly [13]. This algorithm also has a parameter called cost which penalizes misclassification, which is denoted as *C* in the literature. If the data cannot be separated linearly, however, support vector machine can use inner product space also known as kernel to classify the classes [14].

*Neural Network*

Neural network (NN) is a deep learning method that is used for both regression and classification. Despite a long training time to fit a model, it is most suitable in situations in which a dataset has many covariates [2]. A neural network consists of three components: the regressors, the hidden layer, and the outcome. The hidden layer, *Z,* is a linear combination of weights (*w_i_*) extracted from the features and serves as the connection between the regressors and the outcome. It can be expressed mathematically as below.

$$Z=\sum_{i=1}^{N} w_{i}x_{i}$$

Once the hidden layer is established, it projects an outcome depending on the choice of an activation function [8]. A unique feature of this algorithm is backpropagation [15], which involves adjusting the weights from the hidden layer.

*Naïve Bayes*

Naïve Bayes (hereafter NB) is a probabilistic approach to classification which requires conditional independence to compute the probability values [16]. Mathematically, it is represented as the following equation. *X*, $x_{i}$*,* and *n,* indicate the set of all regressors, a single regressor, and the number of regressors.

$$P\left( X | C \right)=\prod_{i=1}^{n} P\left( x_{i} | C \right)$$

$$P\left( x_{i} | C \right)=\frac{P\left( x_{i}\cap C \right)}{P\left( C \right)}$$

While there is no restriction on the probability distribution, normal distribution is often assumed for simplicity of calculations [8]. The main rule of this approach is to select an outcome class with the largest probability given the features.

*Conditional Tree*

Conditional Tree (CT), another tree-based SML, can be used for both regression and classification. Unlike previous tree-based algorithms, CT uses a conditional distribution to build the tree, capturing the relationship between features and outcomes [17,18]. This approach creates a tree by conducting the variable independence test, which examines the conditional distribution and identifies if a statistically significant relationship exists between a particular feature and the outcome. Later, it finds the threshold $t$ that splits a covariate $x_{i}$ into two regions.

*eXtreme Gradient Boosting*

eXtreme Gradient Boosting (XGB) is the “efficient and scalable” version of the gradient boosting algorithm which incorporates forward stagewise additive modeling and tree-based methods [8,19,20]. The algorithm consists of building a tree for either regression or classification while minimizing the loss function generated by the sum of the trees from previous iterations. The key strength of gradient boosting lies in the production of a highly robust tree [19,21]. XGB further improves the tree by implementing additional regularization to both avoid overfitting and achieve better prediction or classification performance [22].

**Evaluation measures**

**Regression (Continuous outcome)**

**Root Mean Squared Error:** Root mean squared error is computed based on the following formula.

$$RMSE=\sqrt{\frac{\sum_{i=1}^{N} (y_{i}-\hat{y}_{i})^{2}}{N}}$$

$N$, $\hat{y}_{i}$, and $y_{i}$ represent the number of observations, the predicted value of the dependent variable after regression, and the actual value of the dependent variable. This measures the error of a statistical prediction model, and it is preferred to have a lower value. In a regression setting, this measure works as the estimator for the standard deviation of the population if there is no bias.

**Mean Absolute Error:** The second measure we use in continuous settings is the mean absolute error ($\mathrm{MAE}$), which is the average absolute value of the prediction. Mathematically, it is calculated as

$$MAE=|\frac{\sum_{i=1}^{N} \left( y_{i}-\hat{y}_{i} \right)}{N}|.$$

While both $\mathrm{MAE}$ and $\mathrm{RMSE}$ are measures of prediction errors, the former does not work as an estimate for the variance of the dependent variable. In general, the former is less than or equal to the latter.

**Coefficient of determination (R^2^):** $R^{2}$ indicates how a small change in the independent variables can explain the changes in the dependent variable. Mathematically, it is expressed as

$$R^{2}=1-\frac{RSS}{TSS}$$

where $\mathrm{RSS}$ and $\mathrm{TSS}$ respectively denote the residual sum of squares and the total sum of squares. The traditional statistics theory suggests using the adjusted $R^{2}$ if there are multiple independent variables in the prediction model. However, most of the machine learning literature only reports the $R^{2}$. Therefore, we do not present the adjusted $R^{2}$ value for this study.

**Classification (Categorical outcomes)**

**Accuracy:** The most important problem in machine learning classification is to have a model that yields the highest value of accuracy. Accuracy indicates the portion that is correctly classified based on a confusion matrix.

| Actual\Predicted | True | False |
| --- | --- | --- |
| True | True Positive (TP) | False Positive (FP) |
| False | False Negative (FN) | True Negative (TN) |

It is therefore the proportion of the diagonal entries of a confusion matrix. Mathematically, it is

$Accuracy=\frac{TP+TN}{TP+FP+FN+TN}$.

**Precision:** Precision specifically looks at instances that are classified as positive. It captures the proportion of instances that are correctly classified as positive. It is also called as the positive predictive value and mathematically takes the following equation.

$Precision=\frac{TP}{TP+FP}$.

**Recall:** Recall looks at the instances that are also positive. However, the difference between precision and recall is that it analyzes the instances that are classified as true positive and false negative. It is also denoted as either the true positive rate or the sensitivity and the mathematical representation is the following.

$$Recall=\frac{TP}{TP+FN}$$

**Specificity:** Specificity is the percentage of true negatives over the sum of true negatives and false positives. A higher specificity indicates that there is a lesser number of false positive observations. Mathematically, it is represented as the following.

$$\mathrm{Specificity}=\frac{TN}{TN+FP}$$

**F_1_-score:** F_1_-score can be regarded as the weighted average of precision recall. It is useful in scenarios in which there is an unbalanced distribution of the instances. The mathematical formula of F_1_-score is the following.

$$\mathbf{F}_{\mathbf{1}}-score=\frac{2*Precision*Recall}{Precision+Recall}$$

**Cohen’s Kappa:** Cohen’s kappa is known as the inter-reliability of the classification. It is computed based on the following five probability values from the confusion matrix. The general rule of thumb is to have a model that yields a kappa value between 0.21 and 1 [23]. A kappa value of 0.21 indicates that a specified classification model does a fair job of categorizing the instances and that of 1 implies a perfect classification performance.

$$p_{0}=Accuracy=\frac{TP+TN}{TP+FP+FN+TN}$$

$$p_{t}=\frac{TP+FP}{TP+FP+FN+TN}\cdot\frac{TP+FN}{TP+FP+FN+TN}$$

$$p_{f}=\frac{FN+TN}{TP+FP+FN+TN}\cdot\frac{FP+TN}{TP+FP+FN+TN}$$

$$p_{e}=p_{t}+p_{f}$$

$$\kappa=\frac{p_{o}-p_{e}}{1-p_{e}}$$

**Area under the curve (AUC):** The last classification measure we look at is the area under the receiver operating characteristic plot, also known as the AUC. Graphically, it shows the trade-off between the false positive and true positive rates at different threshold values. Geometrically, it captures the probability of instances that are correctly specified as either positive or negative. While it is preferred to have a higher value**,** it is important to have it higher than 0.5. A value of 0.5 shows that the model cannot distinguish the two cases correctly.

**References**

1. James G, Witten D, Hastie T, Tibshirani R. An Introduction to Statistical Learning: with Applications in R. New York: Springer; 2013 Jun. doi:10.1007/978-1-4614-7138-7

2. Athey S, Imbens GW. Machine Learning Methods that Economists Should Know About. Annu Rev Econom. 2019;11: 685–725.

3. Tibshirani R. Regression Shrinkage and Selection via the Lasso. Journal of the Royal Statistical Society: Series B (Methodological). 1996 Jan;58(1):267-88. doi:10.1111/j.2517-6161.1996.tb02080.x

4. Wuthrich K, Zhu Y. Omitted variable bias of Lasso-based inference methods: A finite sample analysis. Rev Econ Stat. 2020; 1–47. Available: <http://arxiv.org/abs/1903.08704>

5. Angrist JD, Frandsen B. Machine Labor. J Labor Econ. 2022;40: S97–S140.

6. Saha S, Narayanan S. A simplified measure of nutritional empowerment using machine learning to abbreviate the Women’s Empowerment in Nutrition Index (WENI). World Dev. 2022;154. doi:10.1016/j.worlddev.2022.105860

7. Ahrens A, Hansen CB, Schaffer ME. lassopack: Model selection and prediction with regularized regression in Stata. The Stata Journal. 2020 Mar;20(1):176-235. doi:10.1177/1536867X20909697

8. Hastie T, Tibshirani R, Friedman JH. The Elements of Statistical Learning: Data Mining, Inference, and Prediction. New York: Springer; 2009 Aug. doi:10.1007/978-0-387-21606-5

9. Mullainathan S, Spiess J. Machine learning: An applied econometric approach. Journal of Economic Perspectives. American Economic Association; 2017. pp. 87–106. doi:10.1257/jep.31.2.87

10. Breiman L. Random Forests. Machine Learning. 2001 Oct;45(1):5-32. doi:10.1023/A:1010933404324

11. Schapire RE, Freund Y. Boosting: Foundations and Algorithms. MIT Press; 2012 May 18.

12. Schapire RE. Explaining AdaBoost. Empirical Inference. 2013:37-52. Springer. doi:10.1007/978-3-642-41136-6_5

13. Cortes C, Vapnik V. Support-vector networks. Machine learning. 1995 Sep;20(3):273-97. Doi:10.1007/BF00994018

14. Smola AJ, Schölkopf B. A Tutorial on Support Vector Regression. Statistics and Computing. 2004 Aug;14(3):199-222. doi:10.1023/B:STCO.0000035301.49549.88

15. Mitchell TM. Machine learning. New York: McGraw-Hill; 1997.

16. Han J, Kamber M, Pei J. Data mining: concepts and techniques. Cambridge, MA: Morgan Kaufmann Publishers; 2012.

17. Hothorn T, Hornik K, Zeileis A. Unbiased recursive partitioning: A conditional inference framework. Journal of Computational and Graphical statistics. 2006 Sep 1;15(3):651-74. doi:10.1198/106186006X133933

18. Hothorn T, Hornik K, Zeileis A. ctree: Conditional inference trees. The comprehensive R archive network. 2015;8.

19. Friedman JH. Greedy function approximation: a gradient boosting machine. Annals of statistics. 2001 Oct 1:1189-232. doi:10.1214/aos/1013203451

20. Chen T, He T, Benesty M, Khotilovich V, Tang Y, Cho H, Chen K. Xgboost: extreme gradient boosting. R package version 0.4-2. 2015 Aug 1;1(4):1-4.

21. Friedman JH. Stochastic gradient boosting. Computational statistics & data analysis. 2002 Feb 28;38(4):367-78. <https://doi.org/10.1016/S0167-9473(01)00065-2>

22. Carmona P, Climent F, Momparler A. Predicting failure in the U.S. banking sector: An extreme gradient boosting approach. International Review of Economics and Finance. 2019;61: 304–323. doi:10.1016/j.iref.2018.03.008

23. Landis JR, Koch GG. The Measurement of Observer Agreement for Categorical Data. Biometrics. 1977;33: 159–174.

## **Supplement B. Model performance using 5-fold cross-validation**

In this section, we present the results of model performance using 5-fold cross-validation

**Table B-1. Performance of different SML in predicting BMI using 5-fold cross-validation**

| **Methods** | **R^2^** | **RMSE** | **MAE** |
| --- | --- | --- | --- |
|  | *OOS* | *OOS* | *OOS* |
| LR | 22.1% | 3.404 | 2.774 |
| LLR | 21.7% | 3.412 | 2.783 |
| RT | 15.5% | 3.544 | 2.875 |
| RF | 20.4% | 3.439 | 2.807 |
| CT | 14.9% | 3.558 | 2.890 |
| XGB | 21.7% | 3.411 | 2.777 |
| KNN | 21.9% | 3.416 | 2.779 |
| SVM | 23.9% | 3.372 | 2.718 |
| NN | 14.1% | 3.561 | 2.878 |

Notes: The best performance for each category is in bold. OOS = out-of-sample (test data). Here, LR = Linear regression; LLR = LASSO linear regression; RT = Regression tree; CT= Conditional inference tree; RF = Random forest; XGB = eXtreme Gradient Boosting; KNN = K- nearest neighbor; SVM = Support vector machine; NN = Neural network. *R^2^* = R-squared score for goodness of fit, *RMSE* = Root mean squared error, *MAE* = Mean absolute error.

**Table B-2. Performance of different SML in classifying underweight using 5-fold cross-validation**

| **Methods** | **Accuracy** | | **Sensitivity** | | **Specificity** | | **Precision** | | **Cohen’s** $\boldsymbol{\kappa}$ | | **AUC** | | **F1-Score** | |
| --- | --- | --- | --- | --- | --- | --- | --- | --- | --- | --- | --- | --- | --- | --- |
|  | *IS* | *OOS* | *IS* | *OOS* | *IS* | *OOS* | *IS* | *OOS* | *IS* | *OOS* | *IS* | *OOS* | *IS* | *OOS* |
| LTR | 0.670 | 0.660 | 0.662 | 0.646 | 0.676 | 0.675 | 0.685 | 0.675 | 0.338 | 0.321 | 0.727 | 0.714 | 0.674 | 0.661 |
| LLTR | 0.667 | 0.657 | 0.661 | 0.643 | 0.674 | 0.671 | 0.684 | 0.671 | 0.334 | 0.314 | 0.726 | 0.713 | 0.672 | 0.657 |
| DT | 0.651 | 0.638 | 0.716 | 0.700 | 0.586 | 0.578 | 0.632 | 0.614 | 0.302 | 0.277 | 0.668 | 0.665 | 0.672 | 0.654 |
| RF | 0.956 | 0.876 | 0.986 | 0.954 | 0.926 | 0.801 | 0.930 | 0.821 | 0.920 | 0.752 | 0.994 | 0.963 | 0.957 | 0.883 |
| CT | 0.796 | 0.724 | 0.797 | 0.726 | 0.795 | 0.722 | 0.795 | 0.715 | 0.592 | 0.448 | 0.888 | 0.810 | 0.796 | 0.720 |
| ADB | 0.998 | 0.940 | 0.996 | 0.907 | 0.999 | 0.978 | 0.999 | 0.980 | 0.996 | 0.881 | 0.877 | 0.808 | 0.998 | 0.941 |
| XGB | 0.858 | 0.783 | 0.891 | 0.837 | 0.824 | 0.731 | 0.835 | 0.749 | 0.715 | 0.567 | 0.933 | 0.856 | 0.862 | 0.791 |
| KNN | 0.793 | 0.657 | 0.812 | 0.668 | 0.777 | 0.650 | 0.760 | 0.590 | 0.586 | 0.311 | 0.874 | 0.715 | 0.785 | 0.626 |
| SVM | 0.782 | 0.693 | 0.775 | 0.676 | 0.788 | 0.710 | 0.788 | 0.709 | 0.563 | 0.385 | 0.867 | 0.754 | 0.782 | 0.692 |
| NN | 0.639 | 0.617 | 0.620 | 0.594 | 0.662 | 0.644 | 0.700 | 0.670 | 0.278 | 0.235 | 0.681 | 0.671 | 0.658 | 0.630 |
| NB | 0.647 | 0.646 | 0.638 | 0.631 | 0.656 | 0.662 | 0.673 | 0.665 | 0.294 | 0.292 | 0.707 | 0.700 | 0.655 | 0.648 |

*Notes: IS indicates in-sample (training data) and OOS refers to out-of-sample (test data). Here, LTR= Logistic regression; LLTR = LASSO logistic regression; DT = Decision tree; RF= Random forest; CT = Conditional inference tree; ADB = Adaptive boosting; XGB = eXtreme Gradient Boosting; KNN = K- nearest neighborhood; SVM = Non-linear support vector machine; NN = Neural network; NB = Naïve Bayes. For KNN, SVM, and NN, the data are normalized prior to running the models.*

**Table B-3. Performance of different SML in classifying overweight using 5-fold cross-validation**

| **Methods** | **Accuracy** | | **Sensitivity** | | **Specificity** | | **Precision** | | **Cohen’s** $\boldsymbol{\kappa}$ | | **AUC** | | **F1-Score** | |
| --- | --- | --- | --- | --- | --- | --- | --- | --- | --- | --- | --- | --- | --- | --- |
|  | *IS* | *OOS* | *IS* | *OOS* | *IS* | *OOS* | *IS* | *OOS* | *IS* | *OOS* | *IS* | *OOS* | *IS* | *OOS* |
| LTR | 0.628 | 0.628 | 0.625 | 0.614 | 0.632 | 0.643 | 0.647 | 0.655 | 0.257 | 0.256 | 0.675 | 0.677 | 0.636 | 0.634 |
| LLTR | 0.629 | 0.624 | 0.630 | 0.615 | 0.630 | 0.634 | 0.632 | 0.631 | 0.259 | 0.248 | 0.672 | 0.673 | 0.631 | 0.622 |
| DT | 0.618 | 0.606 | 0.673 | 0.646 | 0.562 | 0.566 | 0.610 | 0.593 | 0.235 | 0.212 | 0.633 | 0.625 | 0.640 | 0.618 |
| RF | 0.931 | 0.805 | 0.935 | 0.835 | 0.927 | 0.775 | 0.927 | 0.782 | 0.862 | 0.610 | 0.980 | 0.892 | 0.931 | 0.808 |
| CT | 0.760 | 0.669 | 0.788 | 0.703 | 0.731 | 0.636 | 0.747 | 0.651 | 0.520 | 0.338 | 0.843 | 0.740 | 0.767 | 0.676 |
| ADB | 0.997 | 0.837 | 0.997 | 0.813 | 0.997 | 0.865 | 0.998 | 0.880 | 0.994 | 0.673 | 0.771 | 0.693 | 0.997 | 0.845 |
| XGB | 0.809 | 0.720 | 0.837 | 0.747 | 0.781 | 0.694 | 0.794 | 0.702 | 0.619 | 0.440 | 0.885 | 0.790 | 0.815 | 0.724 |
| KNN | 0.695 | 0.619 | 0.667 | 0.594 | 0.735 | 0.656 | 0.784 | 0.719 | 0.389 | 0.240 | 0.768 | 0.660 | 0.721 | 0.651 |
| SVM | 0.762 | 0.651 | 0.734 | 0.626 | 0.800 | 0.681 | 0.815 | 0.705 | 0.524 | 0.304 | 0.840 | 0.700 | 0.773 | 0.663 |
| NN | 0.660 | 0.628 | 0.656 | 0.620 | 0.660 | 0.637 | 0.670 | 0.638 | 0.315 | 0.256 | 0.720 | 0.670 | 0.663 | 0.630 |
| NB | 0.621 | 0.619 | 0.619 | 0.610 | 0.623 | 0.630 | 0.632 | 0.632 | 0.242 | 0.238 | 0.664 | 0.672 | 0.626 | 0.620 |

*Notes: IS indicates in-sample (training data) and OOS refers to out-of-sample (test data). Here, LTR= Logistic regression; LLTR = LASSO logistic regression; DT = Decision tree; RF= Random forest; CT = Conditional inference tree; ADB = Adaptive boosting; XGB = eXtreme Gradient Boosting; KNN = K- nearest neighborhood; SVM = Non-linear support vector machine; NN = Neural network; NB = Naïve Bayes. For KNN, SVM, and NN, the data are normalized prior to running the models*

**Table B-4. Performance of different SML in overweight classification based on Asian standard with 5-fold cross-validation**

| **Methods** | **Accuracy** | | **Sensitivity** | | **Specificity** | | **Precision** | | **Cohen’s** $\boldsymbol{\kappa}$ | | **AUC** | | **F1-Score** | |
| --- | --- | --- | --- | --- | --- | --- | --- | --- | --- | --- | --- | --- | --- | --- |
|  | *IS* | *OOS* | *IS* | *OOS* | *IS* | *OOS* | *IS* | *OOS* | *IS* | *OOS* | *IS* | *OOS* | *IS* | *OOS* |
| LTR | 0.585 | 0.564 | 0.625 | 0.607 | 0.545 | 0.521 | 0.580 | 0.551 | 0.170 | 0.130 | 0.618 | 0.595 | 0.578 | 0.578 |
| LLTR | 0.585 | 0.565 | 0.581 | 0.554 | 0.590 | 0.577 | 0.615 | 0.593 | 0.170 | 0.130 | 0.616 | 0.593 | 0.573 | 0.573 |
| DT | 0.575 | 0.567 | 0.628 | 0.622 | 0.520 | 0.513 | 0.570 | 0.553 | 0.150 | 0.135 | 0.589 | 0.573 | 0.586 | 0.586 |
| RF | 0.939 | 0.771 | 0.941 | 0.789 | 0.940 | 0.753 | 0.940 | 0.756 | 0.880 | 0.542 | 0.985 | 0.864 | 0.772 | 0.772 |
| CT | 0.725 | 0.627 | 0.745 | 0.660 | 0.705 | 0.594 | 0.720 | 0.612 | 0.450 | 0.254 | 0.808 | 0.680 | 0.635 | 0.635 |
| ADB | 0.997 | 0.808 | 0.996 | 0.794 | 0.997 | 0.823 | 0.997 | 0.830 | 0.993 | 0.616 | 0.740 | 0.624 | 0.812 | 0.812 |
| XGB | 0.780 | 0.650 | 0.803 | 0.680 | 0.757 | 0.620 | 0.770 | 0.633 | 0.560 | 0.300 | 0.860 | 0.710 | 0.656 | 0.656 |
| KNN | 0.742 | 0.573 | 0.730 | 0.557 | 0.755 | 0.591 | 0.762 | 0.603 | 0.484 | 0.150 | 0.809 | 0.600 | 0.579 | 0.579 |
| SVM | 0.726 | 0.592 | 0.710 | 0.575 | 0.743 | 0.611 | 0.756 | 0.622 | 0.451 | 0.184 | 0.800 | 0.620 | 0.597 | 0.597 |
| NN | 0.570 | 0.570 | 0.553 | 0.543 | 0.600 | 0.612 | 0.700 | 0.711 | 0.142 | 0.142 | 0.592 | 0.600 | 0.616 | 0.616 |
| NB | 0.578 | 0.560 | 0.576 | 0.551 | 0.580 | 0.571 | 0.600 | 0.577 | 0.156 | 0.121 | 0.610 | 0.590 | 0.564 | 0.564 |

*Notes: IS indicates in-sample (training data) and OOS refers to out-of-sample (test data). Here, LTR= Logistic regression; LLTR = LASSO logistic regression; DT = Decision tree; RF= Random forest; CT = Conditional inference tree; ADB = Adaptive boosting; XGB = eXtreme Gradient Boosting; KNN = K- nearest neighborhood; SVM = Non-linear support vector machine; NN = Neural network; NB = Naïve Bayes. For KNN, SVM, and NN, the data are normalized prior to running the models.*

**Table B-5. Performance of different SML in classifying obesity using 5-fold cross-validation**

| **Methods** | **Accuracy** | | **Sensitivity** | | **Specificity** | | **Precision** | | **Cohen’s** $\boldsymbol{\kappa}$ | | **AUC** | | **F1-Score** | |
| --- | --- | --- | --- | --- | --- | --- | --- | --- | --- | --- | --- | --- | --- | --- |
|  | *IS* | *OOS* | *IS* | *OOS* | *IS* | *OOS* | *IS* | *OOS* | *IS* | *OOS* | *IS* | *OOS* | *IS* | *OOS* |
| LTR | 0.702 | 0.685 | 0.692 | 0.668 | 0.714 | 0.704 | 0.727 | 0.711 | 0.405 | 0.371 | 0.767 | 0.752 | 0.709 | 0.689 |
| LLTR | 0.701 | 0.687 | 0.692 | 0.671 | 0.712 | 0.705 | 0.724 | 0.710 | 0.402 | 0.375 | 0.767 | 0.751 | 0.707 | 0.690 |
| DT | 0.700 | 0.679 | 0.757 | 0.741 | 0.625 | 0.620 | 0.668 | 0.652 | 0.382 | 0.359 | 0.705 | 0.700 | 0.709 | 0.693 |
| RF | 0.954 | 0.913 | 0.987 | 0.982 | 0.921 | 0.846 | 0.926 | 0.860 | 0.908 | 0.826 | 0.994 | 0.983 | 0.956 | 0.917 |
| CT | 0.852 | 0.792 | 0.932 | 0.884 | 0.771 | 0.704 | 0.802 | 0.741 | 0.704 | 0.586 | 0.917 | 0.855 | 0.863 | 0.806 |
| ADB | 0.998 | 0.970 | 0.997 | 0.944 | 0.999 | 0.999 | 0.999 | 0.999 | 0.997 | 0.940 | 0.900 | 0.857 | 0.998 | 0.970 |
| XGB | 0.900 | 0.862 | 0.939 | 0.915 | 0.857 | 0.810 | 0.868 | 0.822 | 0.796 | 0.723 | 0.959 | 0.920 | 0.902 | 0.866 |
| KNN | 0.807 | 0.725 | 0.783 | 0.698 | 0.834 | 0.757 | 0.844 | 0.769 | 0.613 | 0.452 | 0.891 | 0.792 | 0.812 | 0.732 |
| SVM | 0.803 | 0.721 | 0.785 | 0.700 | 0.822 | 0.748 | 0.830 | 0.755 | 0.606 | 0.443 | 0.885 | 0.800 | 0.807 | 0.725 |
| NN | 0.724 | 0.696 | 0.718 | 0.688 | 0.729 | 0.703 | 0.728 | 0.686 | 0.447 | 0.391 | 0.800 | 0.762 | 0.723 | 0.687 |
| NB | 0.686 | 0.684 | 0.684 | 0.670 | 0.693 | 0.700 | 0.698 | 0.700 | 0.377 | 0.368 | 0.753 | 0.740 | 0.691 | 0.684 |

*Notes: IS indicates in-sample (training data) and OOS refers to out-of-sample (test data). Here, LTR= Logistic regression; LLTR = LASSO logistic regression; DT = Decision tree; RF= Random forest; CT = Conditional inference tree; ADB = Adaptive boosting; XGB = eXtreme Gradient Boosting; KNN = K- nearest neighborhood; SVM = Non-linear support vector machine; NN = Neural network; NB = Naïve Bayes. For KNN, SVM, and NN, the data are normalized prior to running the models.*

**Table B-6. Performance of different SML in obesity classification based on Asian standard with 5-fold cross-validation**

| **Methods** | **Accuracy** | | **Sensitivity** | | **Specificity** | | **Precision** | | **Cohen’s** $\boldsymbol{\kappa}$ | | **AUC** | | **F1-Score** | |
| --- | --- | --- | --- | --- | --- | --- | --- | --- | --- | --- | --- | --- | --- | --- |
|  | *IS* | *OOS* | *IS* | *OOS* | *IS* | *OOS* | *IS* | *OOS* | *IS* | *OOS* | *IS* | *OOS* | *IS* | *OOS* |
| LTR | 0.666 | 0.671 | 0.665 | 0.662 | 0.668 | 0.678 | 0.664 | 0.663 | 0.333 | 0.341 | 0.737 | 0.741 | 0.665 | 0.663 |
| LLTR | 0.669 | 0.672 | 0.667 | 0.664 | 0.671 | 0.679 | 0.669 | 0.663 | 0.339 | 0.343 | 0.737 | 0.742 | 0.668 | 0.663 |
| DT | 0.660 | 0.666 | 0.620 | 0.616 | 0.700 | 0.713 | 0.672 | 0.672 | 0.321 | 0.330 | 0.672 | 0.674 | 0.645 | 0.643 |
| RF | 0.937 | 0.862 | 0.959 | 0.892 | 0.916 | 0.833 | 0.919 | 0.836 | 0.875 | 0.724 | 0.984 | 0.937 | 0.938 | 0.863 |
| CT | 0.802 | 0.730 | 0.819 | 0.749 | 0.784 | 0.712 | 0.790 | 0.713 | 0.603 | 0.461 | 0.888 | 0.801 | 0.804 | 0.730 |
| ADB | 0.997 | 0.913 | 0.995 | 0.883 | 0.999 | 0.946 | 0.999 | 0.947 | 0.994 | 0.826 | 0.873 | 0.816 | 0.997 | 0.914 |
| XGB | 0.832 | 0.773 | 0.852 | 0.790 | 0.812 | 0.757 | 0.818 | 0.756 | 0.664 | 0.546 | 0.913 | 0.848 | 0.835 | 0.773 |
| KNN | 0.737 | 0.689 | 0.712 | 0.661 | 0.767 | 0.723 | 0.789 | 0.742 | 0.474 | 0.380 | 0.814 | 0.758 | 0.749 | 0.700 |
| SVM | 0.786 | 0.709 | 0.775 | 0.693 | 0.800 | 0.725 | 0.800 | 0.722 | 0.571 | 0.418 | 0.866 | 0.787 | 0.788 | 0.707 |
| NN | 0.693 | 0.674 | 0.696 | 0.667 | 0.690 | 0.680 | 0.677 | 0.659 | 0.385 | 0.347 | 0.764 | 0.742 | 0.686 | 0.663 |
| NB | 0.665 | 0.668 | 0.664 | 0.662 | 0.666 | 0.673 | 0.661 | 0.652 | 0.330 | 0.335 | 0.728 | 0.736 | 0.663 | 0.657 |

*Notes: IS indicates in-sample (training data) and OOS refers to out-of-sample (test data). Here, LTR= Logistic regression; LLTR = LASSO logistic regression; DT = Decision tree; RF= Random forest; CT = Conditional inference tree; ADB = Adaptive boosting; XGB = eXtreme Gradient Boosting; KNN = K- nearest neighborhood; SVM = Non-linear support vector machine; NN = Neural network; NB = Naïve Bayes. For KNN, SVM, and NN, the data are normalized prior to running the models.*

## **Supplement C. Additional figures**

In this section, we present all the supplement figures of this study.


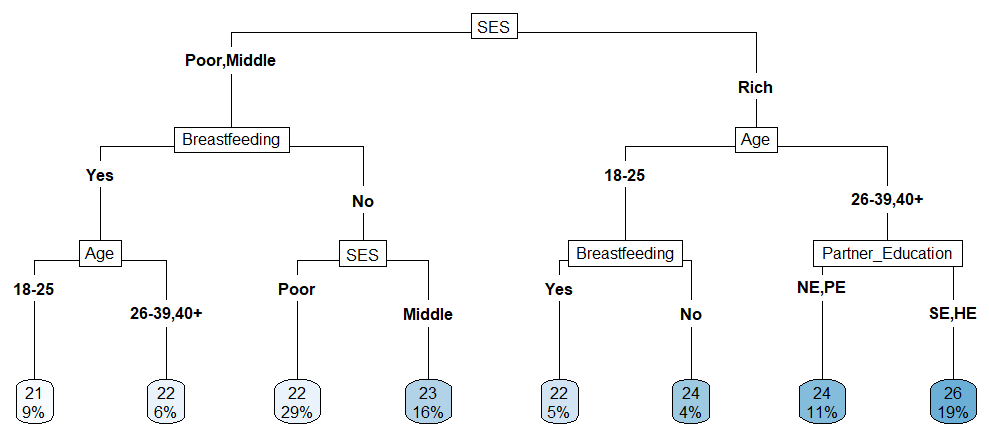


**Fig C-1**: 10-fold cross-validated RT for the prediction of women’s BMI. SES is the acronym for socioeconomic status, and ‘Middle’ indicates middle-class. In women’s age variable, ‘40+’ means at least 40 years of age. In partner’s educational variable, NE, PE, SE, and HE respectively represent no education, primary education, secondary education, and higher degree such as college.


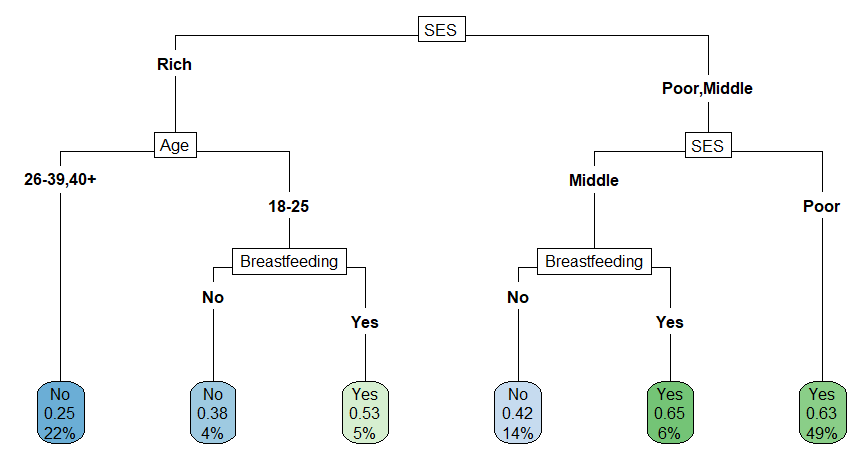


**Fig C-2**: 10-fold cross validated DT for the classification of underweight. SES is the acronym for socioeconomic status, and ‘Middle’ indicates middle-class. In women’s age variable, ‘40+’ means at least 40 years of age.


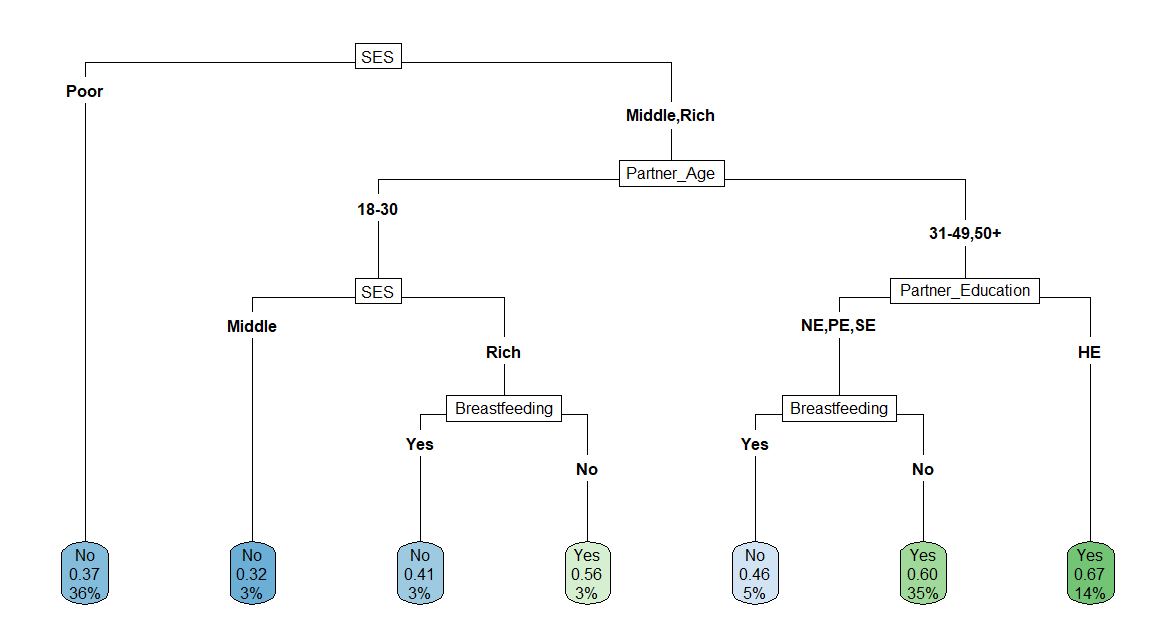


**Fig C-3**: 10-fold cross validated DT for the classification of overweight. ‘50+’ category from partner’s age indicates at least fifty years of age. As seen from the regression tree, NE, PE, SE, and HE respectively represent no education, primary education, secondary education, and higher degree such as college.


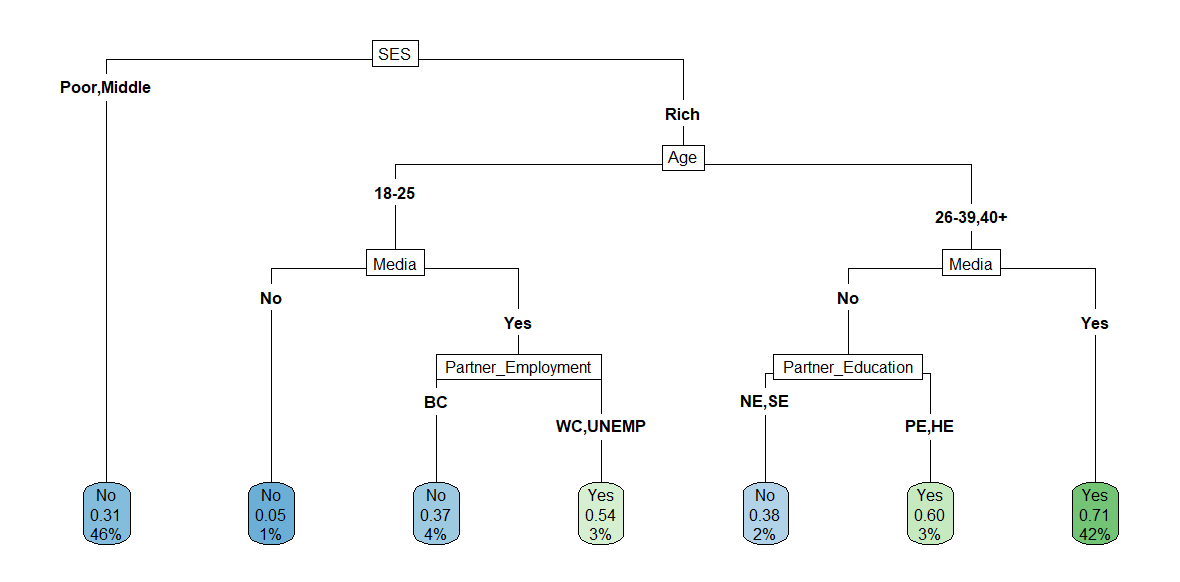


**Fig C-4**: 10-fold cross validated DT for the classification of obesity. BC, WC, and UMEMP categories from the partner’s employment variable respectively represent blue-collar, white-collar, and unemployed.

##

## **Supplement D. Comparative analysis of SML outputs**

**Table D-1. Comparison of SML results between this and previous studies**

| Literature | Outcome | Sample size | Number of features | Algorithm | Accuracy**^†^** | AUC**^†^** | *R^2*^* |
| --- | --- | --- | --- | --- | --- | --- | --- |
| Our study | Underweight | 17,931 | 27 | ADB^1^, RF^2^ | **94.0%**^1^ | **96.2%**^2^ | NA |
|  | Overweight |  |  | ADB^1^, RF^2^ | **84.0%**^1^ | **89.3%**^2^ | NA |
|  | Obesity |  |  | ADB^1^, RF^2^ | 97.0%^1^ | **98.3%**^2^ | NA |
| Ferdowsy et al. [64] | Obesity | 1,100 | 27 | LTR | 97.1% | NA | NA |
| Islam et al. [65] | Underweight | 15,464 | 15 | RF | 81.4% | 83.7% | NA |
|  | Overweight/Obesity |  |  |  | 82.4% | 85.3% | NA |
| DeGregory et al. [57] | Body fat | 25,336 | 6 | NN | NA | 94.0% | NA |
| Figueroa and Flores [61] | Obesity | 66,179 | 500 | SVM | **97.36%** | NA | NA |
| Yadaw et al. [63] | COVID-19 death | 3.841 | 17 | XGB | NA | 91.0% | NA |

Notes: * = continuous outcome metric; ^†^ = categorical outcome metric; ^1^ indicates the accuracy of ADB & ^2^ indicates the AUC value of RF.
