# Supplementary material for: Predicting nutritional status for women of childbearing age from their economic, health, and demographic features: A supervised machine learning approach
Source: PLoS One. 2023 May 12;18(5):e0277738. doi: 10.1371/journal.pone.0277738 (PMC10180666; doi:10.1371/journal.pone.0277738)
Supplement: Attachment — Submitted filename: Response to reviewers.docx [file pone.0277738.s002.docx]

16 April 2023

Dr. Tauseef Ahmad

Academic Editor

PLOS ONE

San Francisco, CA 94111, United States.

**Subject:** Revision of the manuscript PONE-D-22-29868 [EMID:1f09021bb5cd6c6d]

Dear Dr. Ahmad:

We would like to submit the revised version of our research article titled “Predicting nutritional status for women of childbearing age from their economic, health, and demographic features: A supervised machine learning approach.” The revised version addresses all the comments by the reviewers. We have made the necessary changes as instructed in the paper in track changes. We would also like to take the opportunity to thank the reviewers for their insightful comments.

All the authors have approved this revised version of the manuscript and contributed towards the revision. The authors do not have any conflicts of interest to disclose.

Please find the reviewers’ comments and our responses below:

**Comment from Reviewer 1**

1. The value of the wealth index relies on the assets mentioned above owned by households and housing characteristics. However, this study does not suggest asset index as a measure of current welfare or poverty in this paper. Instead, we use it as a determinant of malnutrition among women, which depends on the long run as well as current SES. – this information might have misclassification bias, which was not mentiioned as a limitation.

**Authors’ Response:** We added this as a limitation on p.37, lines 735–741.

2. 5,365 women are removed out of 20,127 from the analysis – there is no explanation for the reason to exclude 5,365 women – this might have selection bias, which was not mentioned as a limitation.

**Authors’ Response:** We explained the exclusions on p.17, lines 370–373. We also added this as a limitation on p.37, lines 741–749.

3. This study evaluates the relationship between categorical features and nutritional outcomes using the Pearson statistic corrected for the complex survey design with the second-order correction of Rao and Scott – This sentence had been presented in the results section and it should be in the methods section.

**Authors’ Response:** We moved the sentence to the methods section (p.17, lines 373–376).

4. There is a statistically significant association between each feature and underweight status, except for abstinence, family planning, and the number of family members. – this sentence is not very clear – it needs to be reviewed.

**Authors’ Response:** We thank the reviewer for highlighting this. We revised the whole section (pp.20–21, lines 443–467) to make this clearer.

5. A mere 5% of the sampled women from high SES are underweight, compared to nearly threefold among those from low socioeconomic backgrounds. – in the result section, no need to use adjective.

**Authors’ Response:** We dropped the adjective and revised the section (pp.20–21, lines 443–467) for clarification.

6. Among breastfeeding women, 16% are underweight compared to 9% among non-breastfeeding women. – no statistic had been presented. And the following comparisons in this section did not present statistical analysis, as well.

**Authors’ Response:** We revised the section (pp.20–21, lines 443–467) for clarification.

**Comments from Reviewer 2**

1. This paper is about Body Max Index and various nutritious rank a set of socioeconomic, health, and demographic features that are critical in predicting nutritional measures several SML feature selection algorithms. The authors have used machine Learning tools which I am not good. Apparently, it looks ok but someone who is expert in Machine learning and artificial intelligence may comment better.

**Authors’ Response:** We thank the reviewer for the careful and insightful review of our manuscript. We used a variety of machine learning algorithms that were used in other health outcome-related studies to derive the results of our study. For instance, Daoud et al. (2019) implemented neural network, linear regression, LASSO regression, random forest, and CART (classification and regression trees) to predict women’s height across 66 nations. DeGregory et al. (2018) attempted logistic regression, decision trees, and neural network to classify the following health outcomes: high blood pressure and body fat. Yadaw et al. (2020) showed that eXtreme Gradient Boosting produced the highest area under the repository curve (AUC) to predict deaths from COVID-19 in the U.S. Rahman et al. (2021) adopted logistic regression, random forest, and support vector machine to predict childhood stunting prevalence in Bangladesh and stated that random forest yielded the best classification results. Lastly, Peterson et al. (2016) used conditional tree analysis to study the association between muscle strength and cardiometabolic risk.

In addition to the algorithms adopted in the aforementioned studies, we also attempted adaptive boosting, k-nearest neighbor, and Naïve Bayes to make our analysis more comprehensive and robust.

References

1. Daoud A, Kim R, Subramanian SV. Predicting women's height from their socioeconomic status: A machine learning approach. Social Science & Medicine. 2019 Oct 1;238:112486.
2. DeGregory KW, Kuiper P, DeSilvio T, Pleuss JD, Miller R, Roginski JW, Fisher CB, Harness D, Viswanath S, Heymsfield SB, Dungan I. A review of machine learning in obesity. Obesity reviews. 2018 May;19(5):668-85.
3. Yadaw AS, Li YC, Bose S, Iyengar R, Bunyavanich S, Pandey G. Clinical features of COVID-19 mortality: development and validation of a clinical prediction model. The Lancet Digital Health. 2020 Oct 1;2(10):e516-25.
4. Rahman SJ, Ahmed NF, Abedin MM, Ahammed B, Ali M, Rahman MJ, Maniruzzaman M. Investigate the risk factors of stunting, wasting, and underweight among under-five Bangladeshi children and its prediction based on machine learning approach. Plos one. 2021 Jun 17;16(6):e0253172.
5. Peterson MD, Zhang P, Saltarelli WA, Visich PS, Gordon PM. Low muscle strength thresholds for the detection of cardiometabolic risk in adolescents. American journal of preventive medicine. 2016 May 1;50(5):593-9.

Sincerely,

Karar Zunaid Ahsan (corresponding author)

Assistant Professor,

Public Health Leadership Program, Gillings School of Global Public Health,

The University of North Carolina at Chapel Hill,

4106 McGavran-Greenberg Hall, 135 Dauer Drive, Chapel Hill, NC 27599-7469, USA.
Email: [zunaid@email.unc.edu](mailto:zunaid@email.unc.edu); Tel: +1 (919) 360-5462
